# Multistaged aortic repair for a 14-year-old girl with Loeys-Dietz syndrome

**DOI:** 10.1016/j.jvscit.2026.102171

**Published:** 2026-02-04

**Authors:** Konstantinos-Eleftherios Koumarelas, Vaiva Dabravolskaite, Florian Schönhoff, Vladimir Makaloski

**Affiliations:** a Department of Vascular Surgery, Inselspital, Bern University Hospital, University of Bern, Bern, Switzerland; b Department of Cardiac Surgery, Inselspital, Bern University Hospital, University of Bern, Bern, Switzerland

**Keywords:** TEVAR, Ductus arteriosus, Loeys-Dietz syndrome, Carotid-subclavian bypass

## Abstract

Loeys-Dietz syndrome is an autosomal-dominant connective tissue disorder characterized by aggressive vascular manifestations, often necessitating early surgical intervention. Informed consent has been obtained from the patient for publication of the case report and accompanying images-videos. Patient gave written informed consent for the anonymous collection of the data for the study in the consent form provided by our institution and approved by Institutional Review Board. We report on a 14-year-old girl with Loeys-Dietz syndrome type 2, with previous open surgical repair of the aortic root and arch, who presented with a symptomatic diverticulum of the ductus arteriosus and critical right upper extremity ischemia, owing to newly diagnosed occlusion of a subclavian artery (SA) aneurysm. Urgent surgical management included the exclusion of the right SA aneurysm using an 8-mm reinforced Dacron graft. In a second procedure 2 days later, left common carotid artery-SA bypass and thoracic endovascular aortic repair landing in zone 2 followed. Postoperative scans revealed distal progression of the disease, resulting in distal extension with thoracic endovascular aortic repair and eventually in an open thoracoabdominal aortic repair.

Loeys-Dietz syndrome (LDS) is a rare, autosomal-dominant genetic disorder that disrupts connective tissue integrity across multiple organ systems. Initially described in 2005, LDS is caused by heterozygous pathogenic variants in genes related to the transforming growth factor beta (TGF-β) signaling pathway, such as *TGFBR1*, *TGFBR2*, *SMAD3*, *TGFB2*, and *TGFB3*, leading to the five types of LDS.^1^ Patients with LDS often present with multisystemic disorder.^1^, ^2^, ^3^, ^4^, ^5^, ^6^ LDS types 1 and 2, caused by mutations in *TGFBR1* and *TGFBR2* respectively, are often associated with pronounced craniofacial features and aggressive vascular involvement. Type 3, linked to *SMAD3*, is distinguished by early-onset osteoarthritis alongside vascular disease. Types 4 and 5, resulting from *TGFB2* and *TGFB3* mutations, tend to present with milder and more variable phenotypes, often with less pronounced craniofacial or skeletal manifestations but still involving aortic pathology. A hallmark of LDS is its aggressive vascular manifestations, including arterial tortuosity, aneurysm formation, and dissection (11%).^1^^,^^5^^,^^7^ These may occur even in childhood and at minor aortic dimensions compared with other heritable thoracic aortic disease (HTAD), like Marfan syndrome (0.8%-12.0%) and/or Ehlers-Danlos syndrome (1%-6%). It leads often to a root replacement before other types of interventions like thoracoabdominal repair, because it usually happens in patients suffering from Marfan syndrome. ^1^^,^^5^^,^^7^ Moreover, the cardiovascular risks associated with LDS, particularly aortic dilation (≥4.0 cm), aneurysms, and dissections, necessitate early interventions depending on the genetic variant and clinical presentation.^1^^,^^2^^,^^4^ Historically, the gold standard for treating these cardiovascular pathologies was an open repair owing to the durability and young patient's age at the time of intervention.^6^ However, promising yet limited results from studies on endovascular or hybrid repair emerge, making a strong argument to address this issue.^8^ In this case report, we present a 14-year-old girl with LDS and two-stage repair of a partially thrombosed right subclavian artery (SA) aneurysm causing upper extremity ischemia and contained rupture of a dissected ductus arteriosus aneurysm. Informed consent has been obtained for publication of the case report and the accompanying images.

## Case presentation

A 14-year-old girl was diagnosed with LDS type 2 (*1570G > T; p.Asp524Asn*, variant in the *TGFBR2* gene). She underwent multiple vascular and orthopedic interventions over a 10-year period, including an aortic root replacement with valve preservation (David procedure) at the age of 5. At the age of 13, she underwent an open aortic arch repair (Gelweave, Terumo Aortic; 26 mm) with distal anastomosis in zone 2, immediately proximal to the left SA (LSA), and separate reimplantation of the brachiocephalic trunk and left common carotid artery (LCCA), performed for an acute type A dissection arising from an aneurysmal ascending aorta with a maximum diameter of 72 mm.^9^, ^10^, ^11^ In addition, she had a history of supraventricular tachycardia, congenital aneurysm of the ductus arteriosus, and recurrent musculoskeletal abnormalities, including a bilateral varus deformity corrected with eight-plate implants.

**Fig 1:**
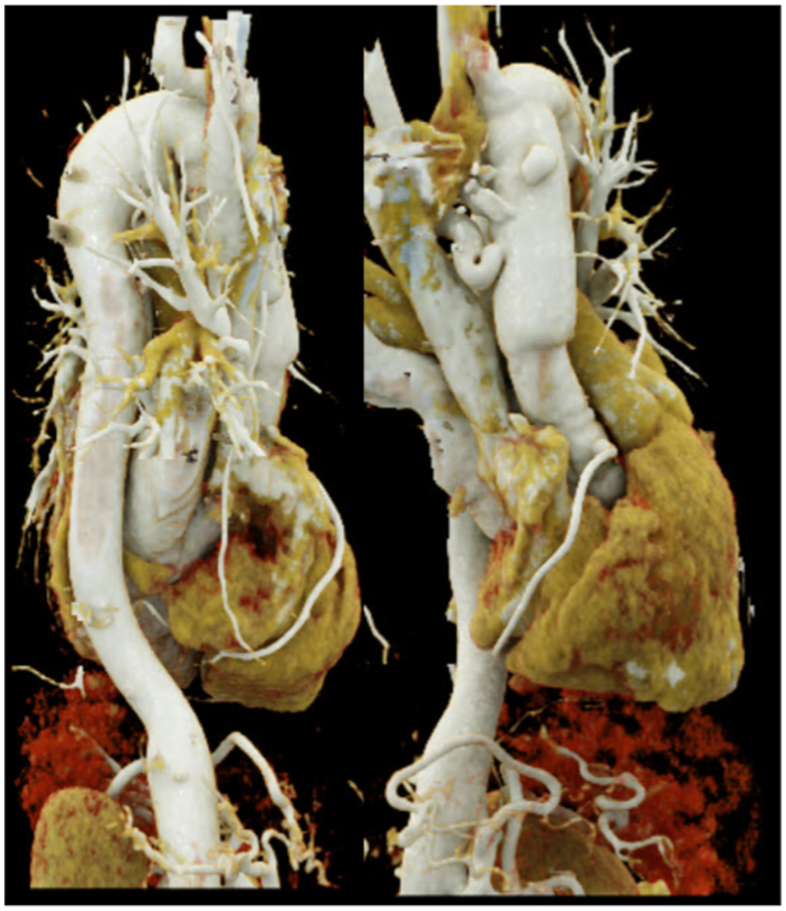
Preoperative three-dimensional reconstruction of the thoracic aorta demonstrating a type III gothic arch configuration. Coronal and sagittal planes are shown for anatomical orientation.

The patient presented to the emergency room with a sudden onset of interscapular pain, which was identical to that experienced during a prior dissection 2 years ago. Upon an initial assessment in emergency room, patient was hemodynamically stable: systolic blood pressure of 140/80 mm Hg, pulse at 77 bpm, respiratory rate at 18/min, oxygen saturation at 97%, and temperature of 36.8 °C, without neurological deficits. Nevertheless, she did not respond to painkillers. An immediate computed tomography angiography (CTA) revealed a contained rupture of a dissected ductus arteriosus diverticulum (9 × 9 × 24 mm) (Supplementary Video 1, online only). Additional enlargement of the thoracic aorta (22 mm) (3 mm in 7 months) (Fig 1), increased diameter of the arch replacement/zone 2 aorta (36.2 mm) and partially thrombosed right SA (RSA) aneurysm with a 21-mm diameter were found. The additional anatomical measurements are 29.7 mm for the ascending aorta, 29.0 mm for the mid aorta, and 26.9 mm for the supraceliac aorta.

After further clinical evaluation and missing right arm pulses, critical ischemia of the right upper extremity was suspected and confirmed by ultrasound examination, showing partial RSA aneurysm thrombosis and occluded brachial artery bifurcation cubital. A multidisciplinary team recommended a staged intervention approach. The upper right extremity circulation was immediately restored by RSA aneurysm exclusion and an 8-mm reinforced Dacron bypass from the proximal RSA to the axillary artery. Both anastomoses were supported with a bovine pericardial strip. Finally, an embolectomy of all arm arteries using a Fogarty catheter via cubital access followed, resulting in palpable radial and ulnar pulses after the operation.

Three days later, a hybrid approach for exclusion of the ductus arteriosus aneurysm was planned. Between the two operations, the patient received antihypertensive therapy, including an angiotensin II receptor blocker and a beta-blocker, as well as analgesics. Anticoagulation was maintained with heparin, targeting an activated clotting time of ≥50 seconds. To create proximal landing zone, an LCCA-LSA bypass using Dacron 8 mm was performed, followed by a thoracic endovascular aortic repair (TEVAR) (Relay, Terumo Aortic; 28 × 28 × 164 mm) implanted via a percutaneous right common femoral artery access, with 8% oversizing. The demanding anatomy, especially caused by the type III gothic aortic arch, led to an unintentional partial coverage of the LCCA (Supplementary Video 2, online only). This was treated with an 8 × 37 mm balloon-expandible chimney stent (BeGraft Plus peripheral, Bentley), through a direct puncture of the distal bypass anastomosis (LCCA). An LSA occlusion with 14 × 10 mm vascular plug (Amplatzer Vascular Plug II) completed the procedure. The postoperative CTA showed completed exclusion of the aneurysm with patent LCCA-LSA bypass.

A follow-up CTA after 1 month showed an endoleak in the arch, most probably originating from the gutters around the LCCA chimney stent, open LCCA-LSA bypass, and excluded aneurysm despite the endoleak (Supplementary Video 3, online only). Four months later, a newly detected distal stent-induced new entry (d-SINE) led to a new onset of back pain, further progression of the dissection in the descending thoracic aorta, and left kidney malperfusion (Supplementary Video 4, online only). The same multidisciplinary team decided to extend immediately the previous thoracic stent graft with two additional thoracic stent grafts (Valiant Captivia, Medtronic; 26 × 26 × 100 distally, above the origin of the celiac trunk and 32 × 28 × 150 mm proximally, connecting the distal thoracic stent graft with the previous one), restoring the left renal perfusion.

**Fig 2:**
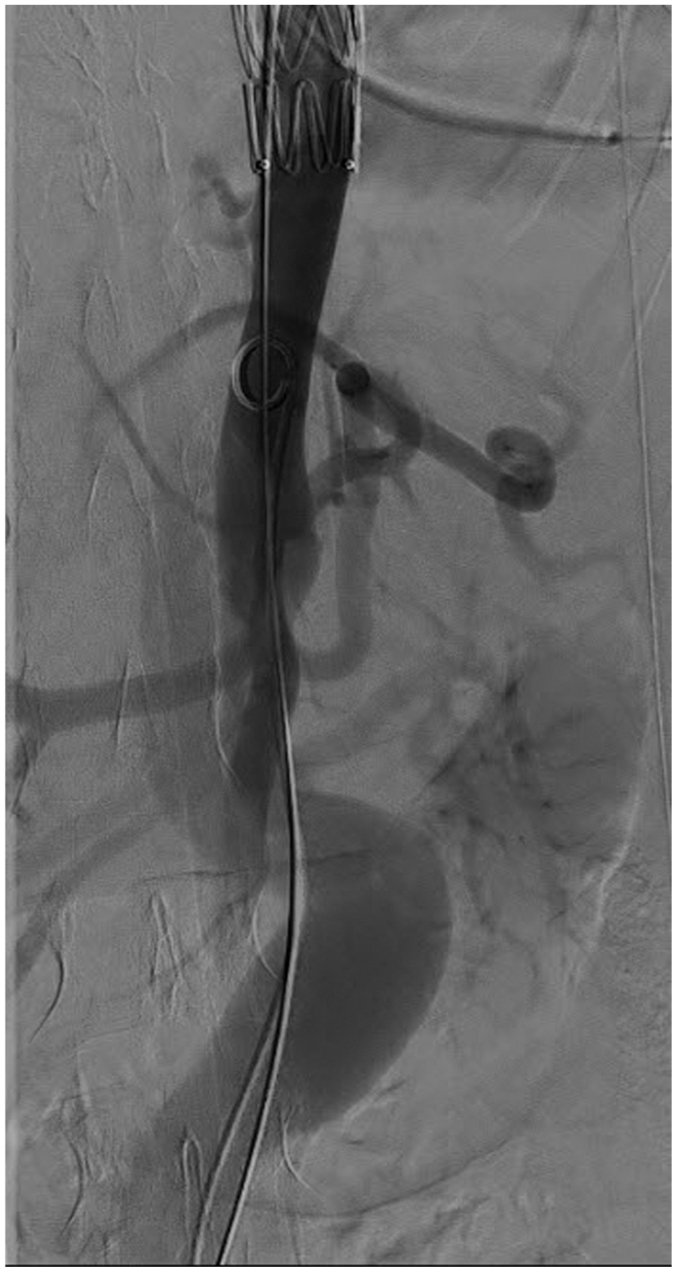
Digital subtraction angiography perioperative showing the highly angulated abdominal aorta.

One month later, follow-up CTA revealed further growth of the dissected descending aorta (53-mm diameter), with a retrograde false lumen perfusion (type Ib endoleak). An additional distal extension was considered to be technically very demanding owing to the massive kinking of the abdominal aorta and the small true lumen, with unclear long-term results in a young LDS patient, owing to the fragile tissue quality associated with LDS (Fig 2). After long discussion with the family, the multidisciplinary team decided to perform an open aortic repair of the thoracoabdominal aorta (Crawford type III procedure) starting from the middle thoracic stent graft and ending infrarenal, with separate reimplantation of all renovisceral arteries. The open repair was performed via a left thoracophrenolumbotomy through the eighth intercostal space and a left-sided cardiopulmonary bypass. Two Cosseli grafts (26 mm Cosseli graft for the thoracic aorta and all branches and 20 mm for the abdominal aorta) were used. The grafts were chosen to match the TEVAR proximally and the smaller distal abdominal aorta accordingly. Neuroprotection measures were also considered, included continuous moto-evoked protentional monitoring selective intercostal artery reimplantation and cerebrospinal fluid drainage. This procedure was uneventful, and the patient was discharged in good general condition. Six months afterward, CTA demonstrated a completely excluded aortic aneurysm with patent supra-aortic and renovisceral arteries. The patient returned to school without any symptoms (Supplementary Video 5, online only).

## Discussion

Patients with LDS require repeated vascular interventions owing to the rapid aneurysmal aortic disease progression.^12^ However, the most common manifestations of the disease lie among the nonaortic vessels (78%).^5^ This report presents a case of a young female LDS patient with multiple previous cardiovascular operations requiring multistage hybrid aortic repair with a good final result.

Open aortic surgery remains the standard treatment of HTAD and aortic pathologies owing to its progressive nature in younger patients.^13^ However, repeated open cardiovascular reinterventions might be a huge physical and psychological burden, even in young patients, diminishing quality of life and advocating for increased hybrid or endovascular solutions. Hybrid operations like frozen elephant trunk have proven beneficial in patients with HTAD, especially in patients with Marfan syndrome suffering an acute aortic dissection. At the same time, they offer a better basis for a second-stage operation.^5^^,^^14^ Frozen elephant trunk is also described as a useful technique for excluding a patent ductus arteriosus.^15^

Despite the benefits, endovascular techniques carry risks such as retrograde dissections and vessel rupture. Olsson et al^16^ observed a primary technical success of 98.2% and a 30-day mortality of 2.9% after treating 171 patients with HTAD endovascularly. The 5-year survival was 80.6% for Marfan syndrome, 85.2% for LDS, and 43.8% for VEDS.^16^ One-half of the patients required secondary procedures, with open conversions in 8.2% after a median follow-up of 4.7 years. Similarly, Cass et al^17^ observed a freedom of open conversion after TEVAR for HTAD of 67.2% at 1 year and 59.7% at 3 years, with the freedom of reintervention being 49.8% and 30.0% at 1 and 3 years, respectively. TEVAR in patients with HTAD also demonstrates a comparable short-term safety to those without HTAD, with similar perioperative mortality (4.6% vs 7.0%), in-hospital complications (26% vs 23%), and 5-year survival rates (81% vs 74%). However, the 2-year reintervention rates were increased in patients with HTAD (25% vs 13%).^18^ In another study from Nucera et al^8^ of 39 patients with HTAD who underwent endovascular aortic repair, the median time to reintervention was 3.9 years (95% confidence interval, 2.0-5.9 years) following planned procedures and 2.0 years (95% confidence interval, −1.1 to 5.1 years) after emergency interventions, although this difference was not statistically significant (*P* = .23). These findings underscore the feasibility and effectiveness of endovascular repair in HTAD patients, despite the high frequency of secondary interventions.^11^ However, a hybrid approach incorporating prior surgical grafts and avoiding postdeployment ballooning has helped to mitigate these complications, providing a safer alternative for complex cases.^12^

In our case, the previous aortic arch replacement served as a landing zone for TEVAR, reducing the risk of proximal fixation site failure. However, it is important to note that the patient had undergone a valve-sparing aortic root replacement. Although valve-sparing aortic root replacement effectively restores physiological flow dynamics in the ascending aorta, it can also lead to postoperative disturbances in the descending aorta, as reported in the literature.^13^ These residual abnormalities—including altered wall shear stress, increased vessel stiffness, and fragility—highlight the need for ongoing surveillance, because they may predispose patients to aneurysm progression or dissection in the distal aorta.^5^^,^^19^ It is also important to note that d-SINE can occur relatively often after TEVAR or fenestrated EVAR, with a percentage varying from 4.4% to 6.5%. Factors like oversizing of the graft and the quality of the vessel play an important role. Owing to the limited off-the-shelf availability, we used a graft with a diameter of 28 mm although the proximal zone was 26 mm and the descending aorta 21 mm (oversizing of 8%).^20^ The new dissection observed in our patient may be linked to these factors. To mitigate the risk of d-SINE, adjunctive strategies have been described, including the use of physician-modified stent grafts, with reduced distal radial force or the implantation of a restrictive distal bare stent to limit the effective TEVAR diameter, both of which have been show decrease the incidence of d-SINE in this high-risk population.^21^^,^^22^

Contained rupture of a ductus arteriosus diverticulum is an exceptionally rare and potentially life-threatening condition, with limited evidence guiding optimal treatment strategies. Management options include open surgical repair with resection or patch repair of the diverticulum, TEVAR with stent graft exclusion, and, in selected cases, endovascular embolization of the diverticular sac. Open surgery provides definitive control but is highly invasive and associated with greater perioperative morbidity, whereas embolization is generally reserved for anatomically unsuitable or high-risk cases. TEVAR is considered the preferred approach when anatomy permits, because it offers effective exclusion of the rupture with lower morbidity and faster recovery, despite the lack of robust evidence owing to the rarity of the condition. In our case, because of the anatomy and the lack of space for the deployment of TEVAR, an LCCA-LSA bypass provided a safe distal landing zone.

Our case presents another key challenge. The patient's young age necessitated careful selection of graft size and type. In younger patients, vessel diameters are likely to expand over time, potentially compromising the durability of the intervention and increasing the risk of complications such as endoleaks and fixation failures.

## Conclusions

This case demonstrates the importance of individualized, multidisciplinary surgical planning in LDS. Given LDS's unpredictable progression, regular imaging and a low threshold for surgical intervention remain essential. Advances in graft materials and molecular therapies may eventually decrease the frequency of surgical interventions for LDS patients. Still, for now, vigilant surveillance and tailored interventions remain the best strategies to manage these high-risk cases effectively.

## Funding

None.

## Disclosures

V.M. reports research and educational grants to the institution from Abbott, Artivion, Biotronik, Boston Scientific, Cook, Medtronic, Nexamedic, Shockwave, and Terumo Aortic. V.M. works as consultant and proctor for Artivion.

## Appendix (online only)

Supplementary Video 1 Covered rupture of the ductus arteriosus and dissection membrane.

Supplementary Video 2 Thoracic endovascular aortic repair (TEVAR) advancement through the gothic arch, with a noticeable jumping motion.

Supplementary Video 3 Postoperative computed tomography (CT) scan showing the carotid-subclavian bypass, thoracic endovascular aortic repair (TEVAR), and complete aneurysm exclusion.

Supplementary Video 4 Newly detected distal stent-induced new entry, progression of the aortic dissection, and left kidney malperfusion.

Supplementary Video 5 Postoperative computed tomography (CT) scan after thoracoabdominal aortic replacement.
